# Polydeoxyribonucleotide and polynucleotide effects in musculoskeletal disorders: a systematic review

**DOI:** 10.3389/fphar.2026.1965494

**Published:** 2026-10-28

**Authors:** Sook-hyun Lee, In-hyuk Ha, Seung Ho Baek, Yoon Jae Lee

**Affiliations:** 1 Jaseng Spine and Joint Research Institute, Jaseng Medical Foundation, Seoul, Republic of Korea; 2 College of Korean Medicine, Dongguk University, Goyang-si, Republic of Korea

**Keywords:** injection, musculoskeletal diseases, polydeoxyribonucleotide (PDRN), polynucleotide, systematic review, tissue regeneration

## Abstract

**Importance:**

The rising prevalence of degenerative musculoskeletal disorders and sports injuries has generated interest in regenerative medicine. Polydeoxyribonucleotide (PDRN) and polynucleotide (PN), extracted from salmonid (Salmonidae) germ-cell DNA, are biomacromolecules with extracellular matrix regenerative, pro-angiogenic, and anti-inflammatory properties.

**Objective:**

We aimed to systematically analyze the clinical and preclinical research trends in PDRN and PN injection therapy in the musculoskeletal field.

**Evidence review:**

Studies on PDRN and PN injection therapy published before March 2026 were retrieved from 10 databases (4 English-, 1 Chinese-, 1 Japanese-, and 4 Korean-language). Clinical studies comprised randomized controlled and clinical studies of adults with musculoskeletal and associated neurological pain; preclinical studies comprised experimental studies using musculoskeletal and related neurological pain models.

**Findings:**

Of the 13,267 records retrieved, 38 (28 clinical and 10 preclinical studies) were included. The clinical studies reported disorders of the knee, shoulder, temporomandibular joint, wrist, ankle/foot, elbow, and spine, reporting overall improvements in pain (visual analog and numerical rating scales) and function (Western Ontario and McMaster Universities Osteoarthritis Index and maximum mouth opening); however, between-group significance varied by disease site and design, particularly with hyaluronic acid comparators. Preclinically, an adenosine A2A receptor-cAMP-PKA-CREB pathway has been proposed, with suppression of M1 and activation of M2 macrophages and upregulation of vascular endothelial growth factor, fibroblast growth factor, and transforming growth factor-β1, promoting type I collagen formation and tendon-to-bone junction reinforcement, while exerting anti-apoptotic effects via caspase-3/9 inhibition. These actions translated into biomechanical outcomes, such as load-to-failure and locomotor recovery. No serious adverse events attributable to PDRN or PN were reported in the available clinical studies; however, this finding should be interpreted cautiously, as adverse event assessment and reporting were incomplete and inconsistent across studies.

**Conclusion and relevance:**

Available evidence suggests potential improvements in pain and function following PDRN/PN treatment; however, the certainty of the evidence remains limited because of methodological limitations, heterogeneous study designs, and inconsistent comparative effects. The limited quality and geographic representativeness of the evidence warrant rigorous, prospectively registered, large-scale, multinational randomized controlled trials.

**Systematic Review Registration:**

https://www.crd.york.ac.uk/PROSPERO/view/CRD420261363033, identifier CRD420261363033 and https://www.crd.york.ac.uk/PROSPERO/view/CRD420261363211, identifier CRD420261363211.

## 1 Introduction

Polydeoxyribonucleotide (PDRN) and polynucleotide (PN) are regenerative treatments for musculoskeletal disorders that promote fundamental tissue recovery via collagen synthesis and angiogenesis. PDRN and PN overcome the adverse effects of corticosteroid injections, offering alternatives for patients experiencing contraindications and adverse effects to corticosteroids (Kim et al., 2024).

PDRN and PN stimulate the intracellular salvage pathway, accelerating DNA synthesis and repair of damaged tissue. By binding to the cell-surface adenosine A2A receptor (A2AR), PDRN exerts anti-inflammatory effects by suppressing inflammatory cytokine secretion and inducing vascular endothelial growth factor (VEGF) expression. A comparable A2AR-mediated action has been proposed for PN, although it has not been directly demonstrated (Hwang et al., 2021; Marques et al., 2025).

Clinical applications of PDRN and PN, extracted from salmon germ cells (*Oncorhynchus keta* or *Oncorhynchus mykiss*), differ owing to molecular weight and physical properties. PDRN, with a relatively low molecular weight of 50–1,500 kDa, is optimized for rapid tissue diffusion and signal transduction and therefore used primarily for tendon and ligament regeneration and amelioration of acute inflammation (Squadrito et al., 2017). PN, which has high-molecular-weight chains exceeding 1,500 kDa, forms a physical three-dimensional scaffold structure *in vivo* (Nam et al., 2025), making it effective for cushioning and cartilage protection in weight-bearing joints such as the knee (Dallari et al., 2020) and useful as a nonsurgical regenerative treatment, that alleviates neural inflammation and induces structural reinforcement of the paraspinal ligaments and muscles in spinal disorders (Irrera et al., 2018; Lee et al., 2021). This structural distinction is also reflected in the regulation. PDRN is classified as a pharmaceutical drug, whereas PN is classified as a medical device in Italy—the country of origin for these compounds (Mastelli Srl)—and South Korea (Agenzia Italiana del Farmaco; Ministry of Food and Drug Safety).

PDRN and PN are increasingly used in clinical practice as promising nonsurgical regenerative treatments for musculoskeletal and neurological disorders (Irrera et al., 2018). Although basic experimental research is underway, systematic reviews that distinguish and define PDRN and PN and comprehensively cover the clinical and preclinical evidence for musculoskeletal applications remain limited (Kim et al., 2024; Luo et al., 2025). A recent narrative review compiled the preclinical and clinical effects of PDRN in tendinopathy, wound healing, and osteoarthritis but lacked a systematic search strategy, study selection criteria, and methodological quality assessment, while addressing PDRN monotherapy only (Kim et al., 2024). Moreover, a scoping review summarized the clinical status of PDRN pharmacopuncture protocols for musculoskeletal disorders and outlined its potential utility in Korean medicine clinical practice (Nam et al., 2025) but incorporated only clinical studies and lacked a translation analysis between the molecular-biological healing mechanisms demonstrated in preclinical models and the clinical effects. Indeed, PN injection is increasingly being used across a range of musculoskeletal conditions, with recent studies reporting its use in hip and shoulder tendinitis (Gervaso et al., 2025); however, such evidence has not yet been systematically synthesized with PDRN and PN clearly distinguished.

In this study, we aimed to systematically analyze the status of domestic and international clinical and preclinical research on the regenerative and analgesic effects of PDRN and PN. By analyzing their potential benefits and safety, we aimed to enhance the clinical utility of PDRN and PN in musculoskeletal and related neurological pain disorders and suggest directions for future research.

## 2 Methods

The study protocol was registered with the International Prospective Register of Systematic Reviews (CRD420261363033 and CRD420261363211). This systematic review complied with the Preferred Reporting Items for Systematic Reviews and Meta-Analyses (PRISMA) guidelines (Moher et al., 2009).

### 2.1 Search strategy

The following databases were searched, without restrictions on language or year of publication: Ovid-Medline, Ovid-EMBASE, Ovid-AMED, and the Cochrane Library (English); China National Knowledge Infrastructure (CNKI; Chinese); CiNii (Japanese); and KoreaMed, Korean Medical Database (KMBASE), Oriental Medicine Advanced Searching Integrated System (OASIS), and Science On (Korean).

The English search terms were confined to words related to or describing the intervention, including “Polydeoxyribonucleotides,” “Polynucleotides,” “PDRN,” and “Placentex.” The search date was March 2026. For the Korean, Chinese, and Japanese databases, the search formulae were modified according to the search algorithms. The search strategy for each database is detailed in Supplementary Table S1.

### 2.2 Inclusion and exclusion criteria

Two authors (SHL and YJL) independently reviewed the titles, abstracts, and full texts of the retrieved results and selected studies for the analysis. Subsequently, they independently verified whether these studies met the eligibility criteria and cross-checked their assessments. Disagreements regarding study selection were resolved consensually by discussion with a third author.

### 2.3 Clinical studies

#### 2.3.1 Types of studies

All randomized controlled and clinical studies using PDRN or PN injection as the intervention were included.

#### 2.3.2 Participants

Adults aged ≥18 years diagnosed with musculoskeletal pain or musculoskeletal-related neurological pain disorders were included, with no restriction on patient population.

#### 2.3.3 Intervention/control

All studies using PDRN or PN injection as the intervention were included, with no restriction on the control group, including studies using hyaluronic acid (HA), corticosteroids, or placebo as the comparator. Studies that did not clearly report the intervention delivery method were excluded.

#### 2.3.4 Types of outcome measures

To evaluate the degree of symptom improvement, pain [visual analog scale (VAS) and numerical rating scale (NRS)] and functional outcomes, among other measures, were included.

### 2.4 Preclinical studies

#### 2.4.1 Types of studies

Randomized and non-randomized experimental studies using PDRN or PN injection as the intervention were included.

#### 2.4.2 Participants

All types of animal models with induced musculoskeletal pain or musculoskeletal-related neurological pain disorders were included. Animal models of other conditions were excluded.

#### 2.4.3 Intervention/control

Studies using PDRN or PN injection as the primary intervention were included. Control groups encompassed placebo, active comparators, or sham-operated controls. Studies using a route of administration other than injection (e.g., oral administration or topical application) or injection of non-PDRN/non-PN agents were excluded.

#### 2.4.4 Types of outcome measures

Studies reporting the biological effects of PDRN or PN and the corresponding mechanisms of action were included; no separate exclusion criteria were applied regarding the outcome measures.

### 2.5 Data extraction

Two authors (SHL and YJL) independently performed data extraction. The following variables were extracted from clinical studies through consensus: principal author, year of publication, study type, diagnostic criteria, sample size, age, sex, intervention/control group information, treatment and follow-up periods, outcomes, and adverse events (Table 1). The following data were extracted from preclinical studies: principal author, year of publication, species/strain, sex, age (or weight), disease/model-induction method, sample size, intervention/control group information, treatment and follow-up periods, outcomes, and adverse events (Table 2). Disagreements were resolved through discussion with a third author.

**TABLE 1:** Summary of clinical studies on PDRN and PN for musculoskeletal and related neurological disorders.

Author (Year/Country)	Study type	Diagnosis, sample size (I/C)	Age, sex (M/F)	Intervention	Control	Treatment period	Follow-up	Outcomes	Adverse effects
A. PDRN monotherapy									
*Temporomandibular joint*									
Choi et al. (2026)/South Korea	Retrospective chart review	TMDs (66; PDRN 30/dextrose 36)	I1: 46.6 ± 17.4 (6/24); I2: 44.8 ± 16.0 (8/28)	I1: PDRN injection (Placentex®/Rejuvenex®/PolyNeo®, 1.0 mL); I2: 10% hypertonic dextrose (1.0 mL)	None	I1: 1–2 weeks intervals; I2: 3 weeks intervals	Mean 2.4 ± 1.0 months	VAS; MMO	Not reported
Jang et al. (2025)/South Korea	Retrospective case series	TMJ-OA (111)	Acute 48.9 ± 13.8 (9/28); Chronic 51.1 ± 16.1 (13/61)	US-guided TMJ pericapsular PDRN (Placentex®, 0.5 mL)	None	2 weeks–1.5 months (mean 3.53 ± 1.16 weeks)	–	MMO; NRS; PGIC	Not reported
*Shoulder joint*									
Do et al. (2018)/South Korea	Clinical trial	Partial-thickness supraspinatus tear (17)	57.9 ± 9.1 (9/8)	US-guided PDRN injection	None	3 times (wk 0, 2, 4)	Wk 0, 6, 12	VAS; DASH; ROM; strength and tear volume (US)	Reported (none)
Ryu et al. (2018)/South Korea	Retrospective chart review	Chronic rotator cuff tendinopathy (131)	53.4 ± 10.0 (17/15)	US-guided PDRN injection	None	3–5 sessions/wk per response	Baseline, 1 week, 1 month, 3 months	VAS; SPADI; SANE	Not reported
Yoon et al. (2017)/South Korea	Retrospective chart review	Rotator cuff disease (106)	I: 54.0 ± 6.6 (25/30); C: 52.4 ± 7.6 (21/30)	PDRN injection	Continued conservative treatment	Weekly intervals, total 3 times	Mo 0, 3, 6	SPADI; VAS	Reported (none)
Kim et al. (2019)/South Korea	Case report	Postherpetic brachial plexopathy (1)	73 (0/1)	PDRN injection	None	3 times, weekly	Mo 6	NRS; motor weakness	Not reported
*Elbow joint*									
Lee and park (2018)/South Korea	Case series	Lateral epicondylitis (2)	65; 59	US-guided PDRN injection	None	2 weeks	Wk 2, mo 2	NRS; Cozen’s test	Not reported
*Wrist joint*									
Park and Park (2018)/South Korea	Case report	Carpal tunnel syndrome (1)	41	US-guided PDRN injection	None	1 week	1 week	NRS; BCTQ	Reported (none)
Hong et al. (2025b)/South Korea	Retrospective case series	Chronic wrist pain (3)	32; 31; 30	US-guided PDRN injection	None	3–4 sessions	–	NRS; joint recess filling	Reported (none)
Hong et al. (2025a)/South Korea	Case report	TFCC injury, wrist pain (1)	29	US-guided PDRN injection	None	3 weeks	–	NRS	Not reported
*Knee joint*									
Mun et al. (2017)/South Korea	Case report	Pes anserine bursitis (1)	50 (0/1)	US-guided PDRN injection	None	Single session	>8 months	NRS	Reported (none)
Kim et al. (2020)/South Korea	Case report	Near-complete ACL tear, partial LCL tear (1)	43 (0/1)	US-/MRI-guided IA PDRN (3 mL PDRN +1% lidocaine)	None	5 times, ∼2 weeks intervals	3 months (up to 2 years 5 months)	NRS; ROM	Reported (none)
*Ankle and foot*									
Kim and Chung (2015)/South Korea	RCT	Chronic plantar fasciitis (40; 20/20)	I: 52 (34–68) (7/13); C: 55 (42–71) (4/16)	PDRN (Placentex®)	Placebo (saline)	3 times, weekly	Baseline, 4, 12 weeks	VAS; MOXFQ	Reported (none)
Lee et al. (2020)/South Korea	RCT	Plantar fasciitis (44)	I: 56.2 ± 12.9 (7/12); C: 50.8 ± 11.5 (2/17)	PDRN 3 mL	Corticosteroid (triamcinolone 20 mg)	3 times, weekly	Baseline, 1, 2, 6 weeks, 6 months	VAS; MOXFQ	Reported (none)
Lim et al. (2016)/South Korea	Case report	Posterior tibial tendon dysfunction (1)	53 (1/0)	US-guided PDRN proliferation therapy (3 mL)	None	3 times, weekly	6 months	NRS; US image	Reported (NSAID-associated edema ∼1 month)
*Spine*									
Kang et al. (2017)/South Korea	Case report	Lumbosacral radiculopathy with type 2 DM (1)	44 (1/0)	Fluoroscopy-guided transforaminal epidural PDRN (5.625 mg/3 mL + 0.3% lidocaine 5 mL)	None	4 times, weekly	>6 months	NRS	Reported (none)
Park (2017)/South Korea	Case report	Traumatic C5 nerve root injury (1)	54 (0/1)	US-guided C5 nerve root block with PDRN (5.625 mg/3 mL)	None	2 times, weekly	2 months	NRS; MMT; CMAP	Not reported
B. PN monotherapy									
*Knee joint*									
Moon et al. (2023)/South Korea	RCT	Knee OA (90; 30/30/30)	I1 67.5 ± 7.0; I2 67.5 ± 10.4; I3 67.0 ± 8.3	PN (IAI, 2 mL, 20 mg/mL; Conjuran)	HA (2 mL, 10 mg/mL); cross-linked HA (3 mL, 20 mg/mL)	3 times, weekly	Baseline, 4, 8, 12, 16, 24 weeks	VAS; K-WOMAC; EQ-5D; K-PDQ	Reported (no severe AEs)
Vanelli et al. (2010)/Italy	RCT	Knee OA (59; 29/30)	I: 60 (37–79) (10/19); C: 67 (46–82)/(10/20)	PN (40 mg/2 mL)	HA (IAI, 16 mg/2 mL)	5 times, weekly	3 months	VAS; KOOS; NSAIDs; crackling & LMA	Reported (none significant)
Kim T. W. et al. (2023)/South Korea	RCT	Knee OA (60; 30/30)	I: 63.6 ± 6.7 (5/25); C: 65.4 ± 8.2	IA PN (Conjuran®)	IA HMWHA (Hyruan Plus)	Baseline, 1, 2 weeks	8, 16 weeks	VAS; K-WOMAC; CGI; PGI; EQ-5D	Reported (2 serious AEs, unrelated)
Giarratana et al. (2014)/Italy	RCT	Knee OA (72; 36/36)	I: 64.9 (20/16); C: 64.1 (15/21)	PN (Condrotide®)	HA (Hyalubrix®)	3 times, weekly	6, 10, 18, 26 weeks	VAS; KOOS; serum COMP	Reported (none significant)
Kim W. H. et al. (2023)/South Korea	Clinical trial	Knee OA (546)	63.5 ± 9.1 (134/412)	PN (Conjuran®)	None	5 times (98.5%)	3, 6 months	VAS; CGI; PGI	Reported (3: 1 mild, 2 moderate; no serious)
Kim et al. (2021)/South Korea	Chart review	Knee OA (15)	PN 68.0 ± 6.4; HA 68.4 ± 8.2; cHA 67.6 ± 6.9	US-guided PN (Conjuran®)	Sodium hyaluronate; cross-linked HA	3 times, weekly	6 weeks	VAS; K-WOMAC; EQ-5D; K-PDQ	Reported (none)
*B. PN monotherapy*									
Mun et al. (2017)/South Korea	Case report	Knee OA (1)	61 (0/1)	US-guided IAI of PN	None	3 times in 3 weeks	>5 months	VAS	Reported (none)
C. PDRN/PN combination therapy									
*Temporomandibular joint*									
Cenzato et al. (2024)/Italy	RCT	TMJ osteoarthrosis (60; 30/30)	I: 51.7 ± 14.6 (8/22); C: 53.3 ± 14.9 (5/25)	PN + HA pericapsular injection	Self-delivered physiotherapeutic exercises	I: 3 times (baseline, 2, 5 weeks); C: 3 times, day	Baseline, 2, 6, 12 weeks	MMO; lateral excursion; VAS	Reported (none)
Elbow joint									
Shim et al. (2021)/South Korea	RCT	Lateral epicondylitis (63)	I1 52.3 ± 10.2 (11/10); I2 52.2 ± 8.2 (13/8); I3 48.7 ± 7.1 (9/12)	EX + PDRN injection (5.625 mg/3 mL)	EX; EX + ESWT	Baseline, 6, 12 weeks	Wk 0, 12	VAS; MEPS; CETD; CDA; CETPTT; CETTT	Not reported
*Knee joint*									
Yoon et al. (2019)/South Korea	RCT	Knee OA (30; 15/15)	I: 65.3 ± 4.8 (7/8); C: 64.9 ± 4.5 (6/8)	PDRN + HA injection	HA injection	3 times, weekly	Baseline, 1, 3, 6 months	VAS; WOMAC; KSS	Reported (no TEAEs/ADRs/and any other complications)
Dallari et al. (2020)/Italy	RCT	Knee OA (100; 50/50)	I: 63.4 ± 6.5 (24/26); C: 64.2 ± 5.1 (22/28)	PN + HA (PN 10 mg/mL co-formulated with HA 40 mg/2 mL)	HA alone (20 mg/2 mL)	3 times, weekly	Baseline, 2, 6, 12 months	KSS; WOMAC; synovial IL-6, IL-8	Reported (none significant)

ACL, anterior cruciate ligament; ADR, adverse drug reaction; AE, adverse event; BCTQ, boston carpal tunnel syndrome questionnaire; CDA, color Doppler activity; CETD, common extensor tendon depth; CETPTT, common extensor tendon partial-thickness tear; CETTT, common extensor tendon tear thickness; CGI, clinical global impression; cHA, cross-linked hyaluronic acid; CMAP, compound motor action potential; COMP, cartilage oligomeric matrix protein; DASH, disabilities of the arm, Shoulder and Hand; DM, diabetes mellitus; EQ-5D, EuroQol 5-Dimension; ESWT, extracorporeal shockwave therapy; EX, exercise; HA, hyaluronic acid; HMW-HA, high-molecular-weight hyaluronic acid; IA, intra-articular; IAI, intra-articular injection; IL, interleukin; KOOS, knee injury and osteoarthritis outcome score; K-PDQ, Korean PainDETECT, questionnaire; KSS, knee society score; K-WOMAC, Korean WOMAC; LCL, lateral collateral ligament; LMA, limitation of articular mobility; MEPS, mayo elbow performance score; MMO, maximum mouth opening; MMT, manual muscle test; MOXFQ, Manchester-Oxford Foot Questionnaire; NRS, numeric rating scale; NSAID, non-steroidal anti-inflammatory drug; OA, osteoarthritis; PDRN, polydeoxyribonucleotide; PGI, patient global impression; PGIC, patient global impression of change; PN, polynucleotide; RCT, randomized controlled trial; ROM, range of motion; SANE, single assessment numeric evaluation; SPADI, shoulder pain and disability index; TEAE, treatment-emergent adverse event; TFCC, triangular fibrocartilage complex; TMD, temporomandibular disorder; TMJ, temporomandibular joint; US, ultrasound; VAS, visual analogue scale; VEGF, vascular endothelial growth factor; WOMAC, Western Ontario and McMaster Universities Osteoarthritis Index.

**TABLE 2:** Summary of preclinical studies on PDRN and PN for musculoskeletal and related neurological disorders.

Author (Year/Country)	Species/Strain/Sex/Age (Weight)	Disease/Model induction	Sample size	Intervention	Control	Treatment period	Follow-up	Outcomes	Adverse effects
A. PDRN monotherapy									
*Lumbar lesion*									
Joo et al. (2023)/South Korea	Rat/Sprague-Dawley/Male/10 weeks (230 ± 10 g)	Sciatic nerve injury; right sciatic nerve compressed with surgical clips (125 g) for 1 min	50 (n = 10/group)	PDRN 2, 4, or 8 mg/kg injected at the injury site once daily	Sham operation; SNI + no treatment	10 days (daily)	10 days post-injury	cAMP, TNF-α, IL-1β, CREB, VEGF, neurofilament; walking-track (SFI), plantar test	Not reported
Lee et al. (2021)/South Korea	Rat/Sprague-Dawley/Male/200–250 g	Lumbar foraminal stenosis; 3-mm stainless-steel rod in left L5 foramen	80 (n = 5–10/group)	Epidural PDRN 5, 8, or 10 mg/kg	Vehicle (epidural 0.9% saline); sham surgery	Single injection, 3 days post-surgery	Up to 21 days (behavioral), 24 days (histology)	von Frey, treadmill; epineurial inflammation, perineurial fibrosis, chromatolysis	Not reported
Lee et al. (2020)/South Korea	Mouse/C57BL/6/Male/adult (25–30 g)	Spinal nerve ligation and chronic post-ischemic pain (neuropathic pain)	∼5–6 per PDRN dose; 4 per vehicle	PDRN 3.3, 10, or 20 mg/kg subcutaneous into ipsilateral hind paw	Vehicle (0.9% NaCl, SC); sham/loose O-ring	8 days	30 min–24 h post-dose	Dose-dependent mechanical allodynia (von Frey); GFAP in DRG and spinal cord	Not reported
*Ankle joint*									
Rho et al. (2020)/South Korea	Rat/Sprague-Dawley/Male/8 weeks (230 ± 5 g)	Achilles tendon injury; ∼50% partial-width surgical excision of right Achilles tendon	40 (n = 8/group)	PDRN 2, 4, or 8 mg/kg injected at the injury site every 2 days (6 injections)	Non-injured control	16 days	Days 6, 11, 15, 16	von Frey, plantar test; H&E; caspase-3/9, Bax, Bcl-2; TNF-α, IL-6, cAMP; CREB, p-CREB, PKA, p-PKA	Not reported
Kang et al. (2018)/South Korea	Rat/Sprague-Dawley/Male/7 weeks (250–300 g)	Achilles tendon injury; full transection of right Achilles tendon with modified Kessler repair	36 (18 exp/18 control; n = 6 per time point)	PDRN 8 mg/kg/day, daily intraperitoneal injection	Normal saline (same volume/route, daily IP)	Daily until sacrifice	1, 2, 4 weeks post-surgery	Biomechanical testing; H&E, collagen I and III, FGF, VEGF, TGF-β1	Not reported
B. PN monotherapy									
*No preclinical study evaluated PN as monotherapy*									
C. PDRN/PN combination or head-to-head comparison									
*Shoulder joint*									
Seo et al. (2024)/South Korea	Rat/Sprague-Dawley/Male/10 weeks (300 ± 5 g)	Chronic rotator cuff tear with diabetes (STZ 65 mg/kg); tendon repaired 4 weeks after infraspinatus detachment	56 (n = 14/group)	PDRN + repair; PN + repair (head-to-head, both with DM)	Saline + repair (±DM); sham (left shoulder)	Repair at wk 5; treatment 2 weeks post-repair (wk 7)	0, 2, 4 weeks post-surgery	CD68, CD168, CSA; load-to-failure, failure mode; VEGF, FGF, IGF-1	Not reported
Hwang et al. (2021)/South Korea	Rat/Sprague-Dawley/Male/300 ± 5 g	Chronic rotator cuff tear; ISP tendon detachment, silicone-hose wrapping, 4-week maintenance	60 (n = 20/group)	PDRN + repair; PN + repair (head-to-head)	Saline + repair (right and left shoulder)	Single injection immediately after surgery	3, 6 weeks post-repair	CD68, CD168, CSA; load-to-failure; collagen continuity/arrangement, fatty degeneration	Not reported
Kim et al. (2021)/South Korea	Rabbit/New Zealand White/Male/12 weeks (skeletally mature)	Rotator cuff tear; 5 × 5 mm full-thickness supraspinatus tear, Penrose tube for 6-week chronicity	32 (n = 8/group)	PDRN alone; PDRN + ESWT; ESWT + PDRN	Normal saline	4 weeks (once weekly)	4 weeks after treatment (total 10 weeks)	PCNA, VEGF, PECAM-1; gross morphology, MT stain, COL-1; motion analysis	Not reported
Kwon and Moon (2020)/South Korea	Rabbit/New Zealand White/Male/2.5–3.0 kg (12 weeks)	Rotator cuff tear; 5 × 5 mm full-thickness tear near subscapularis, silicone tube for 6-week chronicity	24 (n = 8/group)	PDRN + sham MIC; PDRN + MIC	Normal saline	4 weeks	Immediately after 4-week treatment (total 10 weeks)	Gross morphology; histologic MT score; PCNA, VEGF, PECAM-1; motion analysis	Not reported
*Ankle joint*									
Kim et al. (2025)/South Korea	Rat/Sprague-Dawley/Male/8 weeks (250–300 g)	Achilles tendon injury; 1.5-mm punch-biopsy defect in right Achilles tendon	32 (n = 8/group)	PDRN (0.375 mg); atelocollagen (6 mg); PDRN + atelocollagen	Normal saline (0.2 mL)	Single injection, 1 day after injury	1 and 4 weeks	Biomechanical testing; neutrophil count, inflammatory index, collagen, fibroblast; collagen I, VEGF, TGF-β1, basic FGF	Not reported

Bax, Bcl-2-associated X protein; Bcl-2, B-cell lymphoma 2; cAMP, cyclic adenosine monophosphate; CD68/CD168, cluster of differentiation 68/168; COL-1, collagen type I; CPIP, chronic post-ischemic pain; CREB, cAMP, response element-binding protein; CSA, cross-sectional area; DM, diabetes mellitus; DRG, dorsal root ganglion; ESWT, extracorporeal shockwave therapy; FGF, fibroblast growth factor; GFAP, glial fibrillary acidic protein; H&E, hematoxylin and eosin; IGF-1, insulin-like growth factor 1; IL-1β/IL-6, interleukin-1β/6; IP, intraperitoneal; ISP, infraspinatus; MIC, microcurrent therapy; MT, Masson’s trichrome; PCNA, proliferating cell nuclear antigen; p-CREB, phosphorylated CREB; PECAM-1, platelet endothelial cell adhesion molecule-1; PDRN, polydeoxyribonucleotide; PKA, protein kinase A; PN, polynucleotide; p-PKA, phosphorylated PKA; SC, subcutaneous; SFI, sciatic functional index; SNI, sciatic nerve injury; SNL, spinal nerve ligation; STZ, streptozotocin; TGF-β1, transforming growth factor-β1; TNF-α, tumor necrosis factor-α; VEGF, vascular endothelial growth factor.

### 2.6 Risk of bias assessment

Two authors (SHL and YJL) independently assessed the methodological quality (risk of bias) of the included studies, with disagreements resolved as above. Because the included studies differed in design, design-specific appraisal tools were applied. For clinical studies, randomized controlled trials were assessed using the revised Cochrane risk-of-bias tool (RoB 2) (Sterne et al., 2019); non-randomized comparative studies using the Risk Of Bias In Non-Randomized Studies - of Interventions (ROBINS-I) (Sterne et al., 2016); single-arm before–after studies using the National Heart, Lung, and Blood Institute Quality Assessment Tool for Before-After (Pre-Post) Studies With No Control Group (National Heart, Lung, and Blood Institute, 2014); case series using the Joanna Briggs Institute (JBI) Critical Appraisal Checklist for Case Series (Munn et al., 2019); and single-patient case reports using the JBI Critical Appraisal Checklist for Case Reports (Moola et al., 2024). For comparisons supported by controlled evidence, the certainty of evidence for pain outcomes was rated per comparison using the Grading of Recommendations Assessment, Development, and Evaluation (GRADE) approach (Guyatt et al., 2008). For preclinical studies, risk of bias was assessed using the Systematic Review Centre for Laboratory Animal Experimentation (SYRCLE) risk-of-bias tool (Hooijmans et al., 2014).

### 2.7 Data synthesis

As the included studies were clinically and methodologically heterogeneous, a meta-analysis was not performed; instead, a qualitative synthesis was conducted. Studies were grouped and compared according to the following criteria: intervention agent (PDRN vs. PN); monotherapy versus combination therapy; anatomical site or clinical condition; type of comparator; study design; outcome domain; and follow-up duration. For interpretation, findings were summarized separately according to the strength of the underlying design, distinguishing (i) controlled comparative evidence (randomized and non-randomized), (ii) uncontrolled single-arm before–after evidence, and (iii) case reports and case series. Within-group improvement from baseline was not interpreted as equivalent to a between-group treatment effect. Where available, effect estimates and their precision (e.g., mean changes with standard deviations or confidence intervals) were reported rather than relying solely on statistical significance; however, as many studies did not report effect sizes or precision, the quantitative synthesis was limited.

## 3 Results

### 3.1 Search results

The database search yielded 13,267 records as follows: MEDLINE (n = 9,790), EMBASE (n = 2,979), AMED (n = 1), Cochrane (n = 175), CNKI (n = 100), CiNii (n = 30), KoreaMed (n = 52), KMBASE (n = 6), OASIS (n = 7), and Science On (n = 127). After eliminating duplicates, 10,023 records underwent title/abstract screening, of which 9,909 irrelevant studies were excluded (Figure 1). The remaining 114 studies underwent a full-text review, leading to the exclusion of 76 reports that were not preclinical/clinical studies or for other reasons. Finally, 38 studies (28 clinical and 10 preclinical) (Figure 1) published between 2010 and 2026 were included.

**FIGURE 1:**
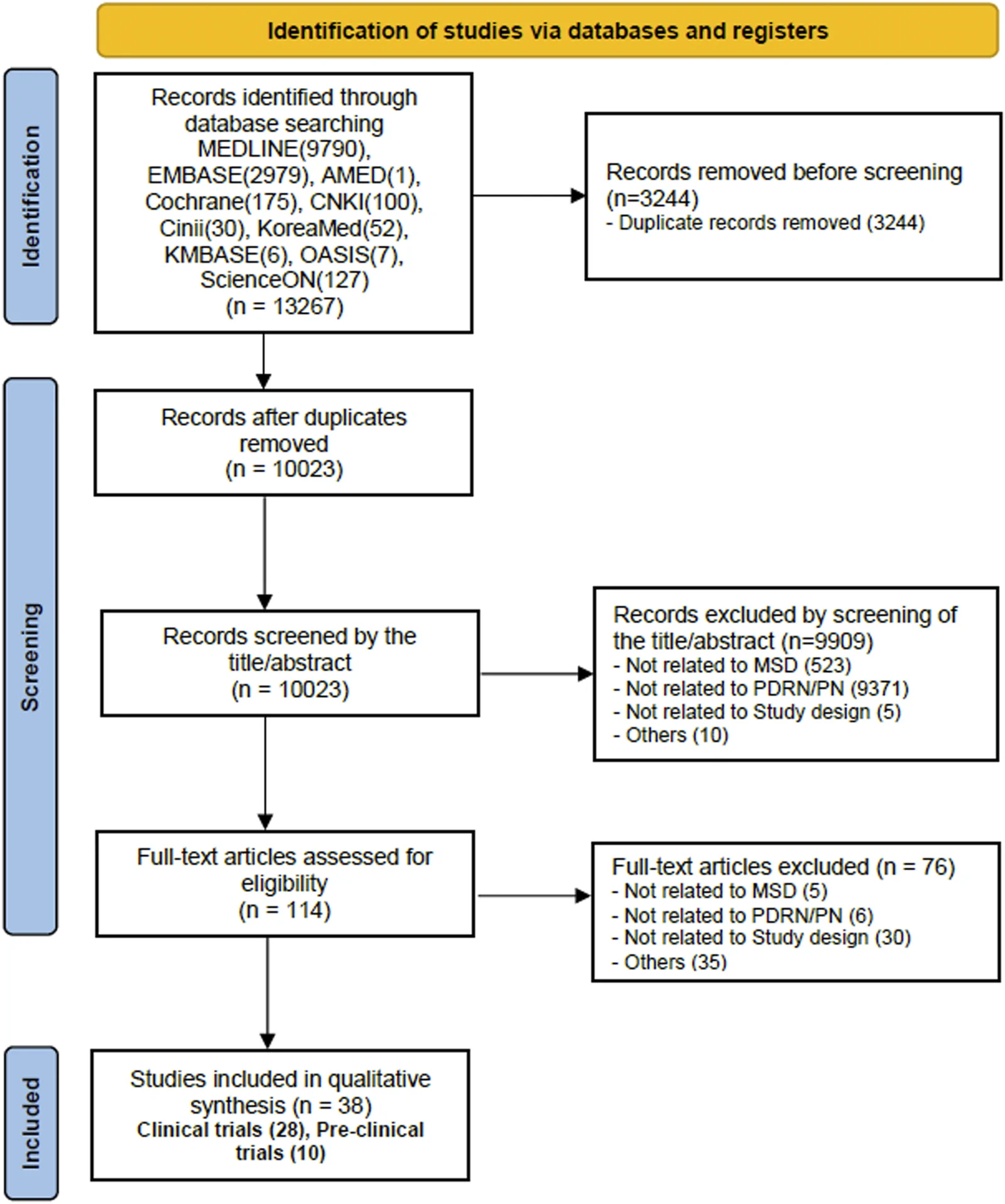
Flow chart of the study selection process.

### 3.2 Included studies

The studies analyzed in this review were categorized as clinical and preclinical (Tables 1, 2). The 28 clinical studies targeted patients with musculoskeletal and related neurological pain. Knee disorders were the predominantly investigated musculoskeletal condition (11 studies: knee osteoarthritis, n = 9; pes anserine bursitis, n = 1; anterior cruciate ligament/lateral collateral ligament tear, n = 1), followed by shoulder (4 studies: rotator cuff, n = 3; postherpetic brachial plexopathy, n = 1), temporomandibular joint (TMJ) (3 studies: TMJ osteoarthrosis, n = 2; temporomandibular disorder, n = 1), wrist (3 studies: carpal tunnel syndrome, n = 1; wrist pain, n = 1; triangular fibrocartilage complex injury wrist pain, n = 1),elbow (lateral epicondylitis, n = 2), ankle/foot (3 studies: plantar fasciitis, n = 2; posterior tibial tendon dysfunction, n = 1), and spinal disorders (2 studies: lumbosacral radiculopathy, n = 1; traumatic C5 nerve root injury, n = 1).

The 10 preclinical studies reported investigations of shoulder (rotator cuff tear, n = 4), lumbar (sciatica, n = 1; lumbar foraminal stenosis, n = 1; neuropathic pain, n = 1), and ankle disorders (Achilles tendon injury, n = 3).

PDRN/PN interventions were administered via various modes of injection and as monotherapy or as part of combination therapy. Most clinical studies reported the use of ultrasound-guided injection. For the TMJ, PN + HA, PDRN alone, and ultrasound-guided PDRN injection were performed; for the shoulder and wrist, PDRN injection (including ultrasound-guided) predominated. For the elbow, PDRN was combined with exercise therapy and delivered via ultrasound-guided injection. For the knee, PDRN, PN, and PDRN + HA combination therapy and ultrasound-guided injection were administered via different routes; PDRN injection was mainly used for the ankle/foot. For spinal disorders, fluoroscopy-guided epidural PDRN injection was performed for lumbosacral radiculopathy, and ultrasound-guided nerve root block was administered for cervical nerve root injury. In preclinical studies, PDRN or PN combined with another intervention was delivered in animal shoulder disorder models. In the lumbar and ankle disorder models, all studies used PDRN as monotherapy.

The control groups were categorized into active and placebo. The control groups in clinical studies differed according to the characteristics of each disorder. For TMJ disorders, active interventions, such as physiotherapeutic exercises, constituted the control, and active interventions, such as exercise therapy and extracorporeal shockwave therapy, were used for elbow disorders. For knee disorders, HA injections were used as the control in 7 studies. For ankle/foot disorders, corticosteroid or saline injection was used as the control.

In most preclinical studies, saline was used as the control to facilitate comparison of the intervention effects (shoulder, n = 4; lumbar, n = 2; ankle, n = 2).

### 3.3 Quality assessment of included studies

#### 3.3.1 Clinical studies

Quality assessment was performed using the Cochrane Risk of Bias (RoB) 2 tool for the 10 clinical RCTs included in this systematic review. For bias arising from the randomization process (D1), most studies were rated “low risk” regarding random sequence generation, achieved using computerized randomization programs, Excel-based block randomization, or randomization envelopes; however, because the allocation concealment method was not specified, five studies were ultimately classified as having “some concerns,” and one study was rated as “high risk.” For bias due to deviations from the intended intervention (D2), seven studies were rated as “low risk,” whereas one study was rated as “high risk” owing to the absence of a specific allocation concealment method and significant baseline imbalance, and two other studies were rated as having “some concerns” because they used a single-blind design wherein patients and operators were aware of group allocation, and the relevant description was insufficient. For bias due to missing outcome data (D3), eight studies were rated “low risk,” whereas one study was rated as “high risk” because the high dropout rate encumbered intention-to-treat analysis, and one study was classified as having “some concerns” because this was not specifically described. For bias in the measurement of the outcome (D4), most studies had a low RoB; however, three studies were rated as having “some concerns” because they lacked a specific description of the subjective outcome and administered self-report questionnaires under conditions where patient blinding was difficult. Conversely, even for subjective questionnaire outcomes, studies wherein the blinding of patients, operators, and outcome assessors was rigorously maintained and managed were rated as “low-risk.” For bias in the selection of the reported result (D5), three studies were rated as “low risk,” whereas the remaining studies, for which protocol registration could not be verified, were classified as having “some concerns.” Integrating the overall RoB of the included studies, one study was rated as “low risk,” two studies as “high risk,” and the remaining studies had “some concerns” (Figure 2). Using ROBINS-I, both non-randomized comparative studies were judged to have a serious overall risk of bias, driven mainly by uncontrolled confounding. Using the National Institutes of Health (NIH) tool, both single-arm before–after studies were rated as fair. According to the JBI checklists, the case series were generally of adequate methodological quality, although uncertainty persisted regarding the consecutive or complete inclusion of participants. All single-patient case reports met the majority of the criteria presented in Supplementary Table S2.

**FIGURE 2:**
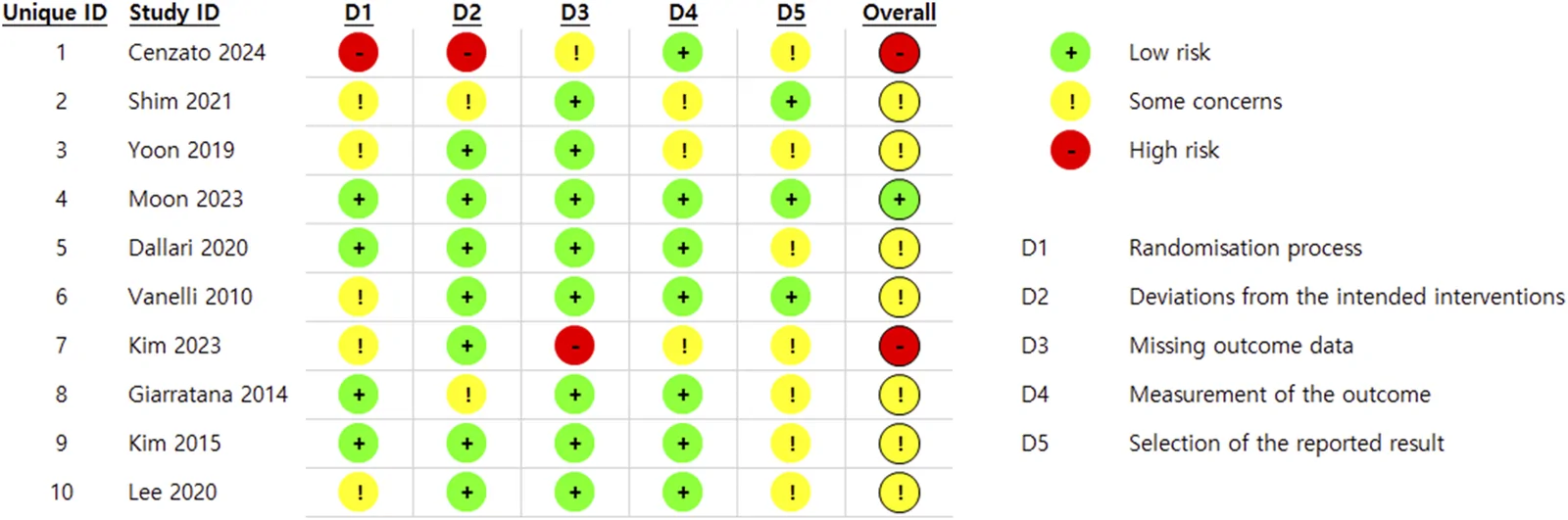
Risk of bias assessment of included studies (Risk-of-Bias 2 tool).

#### 3.3.2 Preclinical studies

The SYRCLE risk‐of-bias tool revealed distinct differences in risk by assessment item in the 10 preclinical RCTs in this systematic review. Within the selection bias domain, baseline homogeneity (D2) was rated as “low risk” in all included studies, demonstrating the uniformity of the experimental animal models. However, sequence generation (D1) and allocation concealment (D3) were classified as “unclear” in most studies because the randomization method was not specified. In the performance bias domain, random housing (D4) and blinding of investigators (D5) were rated “unclear” across all studies because they were not categorically mentioned by any study. Conversely, incomplete outcome data (D8) in the attrition bias domain, selective outcome reporting (D9) in the reporting bias domain, and other sources of bias (D10) were all rated as “low risk,” as they were reported transparently and without missing data across all studies. Finally, in the detection bias domain, random outcome assessment (D6) was rated as “unclear” in most studies, except one, owing to an inadequately detailed description, whereas for blinding of the outcome assessor (D7), 6 studies (excluding 4) reported blinding and were classified as “low risk” (Figure 3).

**FIGURE 3:**
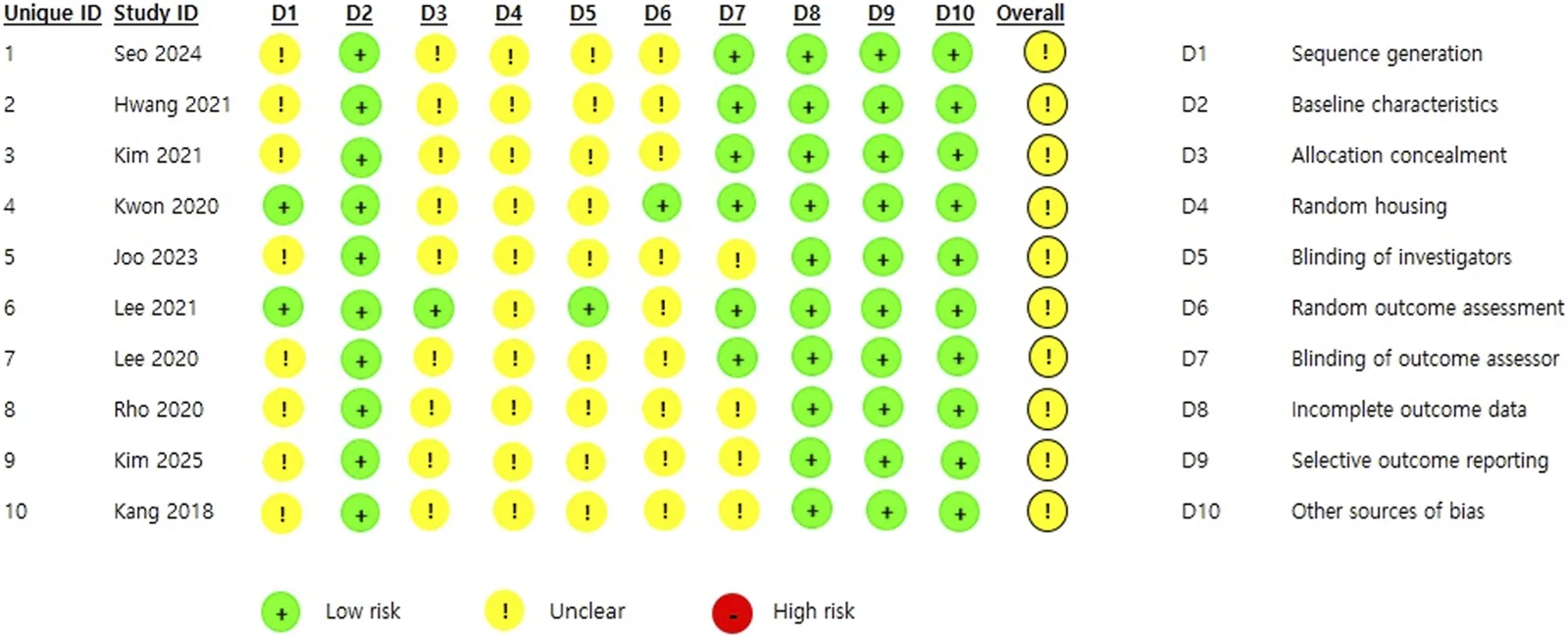
Risk of bias assessment of animal studies (SYRCLE tool).

### 3.4 Effectiveness

#### 3.4.1 Clinical effects of PDRN/PN

The clinical effects of the PDRN/PN intervention were evaluated using outcome measures such as pain (VAS, NRS), functional disability [Western Ontario and McMaster Universities Osteoarthritis Index (WOMAC), maximum mouth opening (MMO)], and quality of life [EuroQol 5-Dimension (EQ-5D)].

Regarding pain improvement by disease site, one study reported that PN + HA pericapsular injection for TMJ disorders produced a statistically significant improvement in VAS pain compared with physiotherapy (Cenzato et al., 2024), and one case series demonstrated greater pain improvement in the acute group than in the chronic group (Jee-Won Jang et al., 2025). In a prolotherapy study reporting a comparison between PDRN and hypertonic dextrose solution, VAS scores demonstrated pain amelioration, but the between-group difference lacked statistical significance (Choi et al., 2026). One study reported measurement of the MMO, showing nonsignificant improvement in maximum opening, albeit with a significant improvement in left–right asymmetry (Cenzato et al., 2024). Two other studies also reported an MMO improvement effect, albeit without a significant difference compared with the control (Jee-Won Jang et al., 2025; Choi et al., 2026).

For shoulder disorders, one study reported a comparison between PDRN injection and continued conservative treatment and reported a significant between-group difference in pain improvement (Yoon et al., 2017), whereas three studies reported significant pain improvement compared with baseline (Do et al., 2018; Ryu et al., 2018; Kim et al., 2019). For elbow disorders, two studies reported pain improvement (Lee and Park, 2018; Shim et al., 2021). For wrist disorders, all three studies using ultrasound-guided PDRN injection reported consistent improvement in pain (Park and Park, 2018; Hyeon Joon Hong, 2025a; Hyeon Joon Hong, 2025b).

Among lower-extremity joint disorders, four studies reported significant between-group differences in knee pain improvement: the PDRN or PDRN + HA injection group was superior to the HA injection group (Yoon et al., 2019), the PN group was superior to the classic HA and crosslinked HA groups (Moon et al., 2023), and the PNHA treatment group was superior to the HA treatment group (Dallari et al., 2020), each showing significantly superior pain improvement. The remaining studies reported pain improvement without a significant between-group difference relative to the control (Giarratana et al., 2014; Mun et al., 2017a; Mun et al., 2017b; Kim et al., 2020; Kim W. H. et al., 2023); among these, in the study comparing PN with HA, the PN group showed a greater reduction in pain versus the HA group, particularly weight-bearing knee pain (Kim J. Y. et al., 2021).

For ankle/foot disorders, one study reported that PDRN injection produced a pain improvement effect similar to that of corticosteroid injection at the 6-month follow-up (Lee et al., 2020). In one study of plantar fasciitis, the PDRN group showed no significant between-group difference from the placebo group at 4 and 12 weeks, but showed significantly greater pain improvement compared with the placebo group after 12 weeks of treatment (Kim and Chung, 2015). In another study, ultrasound-guided prolotherapy for ankle ligament injury elicited pain amelioration (Lim et al., 2016).

Across the included studies, PDRN/PN was generally associated with within-group reduction in pain from baseline. Some knee and TMJ trials reported greater reduction than that observed with hyaluronic acid, saline, or physiotherapy, whereas no consistent difference was observed with more active comparators such as crosslinked hyaluronic acid or corticosteroids. However, these findings should be interpreted cautiously, as randomized trials were largely confined to the knee and foot, whereas shoulder, wrist, and spine data were derived mainly from case reports or small series (Table 3).

**TABLE 3:** Pain outcomes of PDRN and PN interventions across musculoskeletal and related neurological disorders.

Author (Year/Country)	Study type	Site/Diagnosis	Pain measure	Intervention	Comparator	Baseline pain	Endpoint pain	Within-group change	Between-group significance (vs. comparator)
A. PDRN monotherapy									
*Temporomandibular joint*									
Choi et al. (2026)/South Korea	Retrospective chart review	TMDs	VAS	PDRN injection	10% hypertonic dextrose (active comparator; another proliferant, not placebo)	4.70 ± 1.78 (PDRN group)	0.98 ± 1.21 (final follow-up)	Significant reduction (p < 0.001)	No significant difference vs. dextrose (final VAS p = 0.936); both agents improved significantly
Jang et al. (2025)/South Korea	Retrospective case series	TMJ-OA	NRS	US-guided pericapsular PDRN injection	None (single-arm)	4.36 ± 2.35 (overall)	1.48 ± 1.86 (final follow-up)	Significant reduction (acute ΔNRS −3.74; chronic −2.64)	N/A — single-arm case series
*Shoulder joint*									
Do et al. (2018)/South Korea	Clinical trial (pilot)	Partial-thickness supraspinatus tear	VAS	US-guided PDRN injection	None (single-arm)	5.53 (active); 3.71 (resting)	3.53 (active, 12 weeks); 2.71 (resting)	Active VAS significant (p = 0.016); resting NS (p = 0.168)	N/A — single-arm open-label pilot
Ryu et al. (2018)/South Korea	Retrospective chart review	Chronic rotator cuff tendinopathy	VAS	US-guided PDRN injection	None (single-arm)	5.3 ± 1.2	1.7 ± 1.3 (3 months)	Significant reduction at all timepoints (p < 0.001)	N/A — single-arm chart review
Yoon et al. (2017)/South Korea	Retrospective chart review	Rotator cuff disease	VAS	PDRN injection	Continued conservative treatment	5.8 ± 0.9 (month 0)	4.1 ± 1.6 (month 6); 3.9 at month 3	Significant time effect (p < 0.001); M0→M3 −1.9 (> MCID 1.4)	Significant only at month 3 (p = 0.001); NS at months 0 and 6
Kim et al. (2019)/South Korea	Case report	Postherpetic brachial plexopathy	NRS	PDRN injection	None (single-arm)	9 (pre-injection)	3 (sustained at 6 months)	Marked reduction (9→4 within 1 day; sustained at 3)	N/A — single-patient case report
*Elbow joint*									
Lee et al. (2018)/South Korea	Case series	Lateral epicondylitis	NRS	US-guided PDRN injection	None (single-arm)	Case 1: 7; Case 2: 7 (pre-injection)	Case 1: 1; Case 2: 2 (2 weeks)	Significant relief (Case 1–6; Case 2–5)	N/A — 2-patient case series
*Wrist joint*									
Park and Park, (2018)/South Korea	Case report	Carpal tunnel syndrome	NRS	US-guided PDRN injection	None (single-arm)	7 (pre-injection)	2 (6-month follow-up)	Marked reduction (7→2, sustained); BCTQ improved	N/A — single-patient case report
Hong et al. (2025b)/South Korea	Retrospective case series	Chronic wrist pain	NRS	US-guided PDRN injection	None (single-arm)	Case 1: 6; Case 2: 5; Case 3: 6–7	Case 1–3: 0–1 (after 3–4 sessions)	Marked short-term reduction in all three, esp. Weight-bearing	N/A — 3-case series
Hong et al. (2025a)/South Korea	Case report	TFCC injury, wrist pain	NRS	US-guided PDRN injection	None (single-arm)	5 (weight-bearing; initial 6)	0 (after 4 sessions)	Gradual reduction (5→0); improved ROM	N/A — single-patient case report
*Knee joint*									
Mun et al. (2017)/South Korea	Case report	Pes anserine bursitis	NRS	US-guided PDRN injection	None (single-arm)	7 (unable to walk; NSAID/PT-refractory)	0 (2-week follow-up; sustained >8 months)	Gradual reduction (−7; no test, n = 1)	N/A — single-patient case report
Kim et al. (2020)/South Korea	Case report	Near-complete ACL/partial LCL tear	NRS	US-/MRI-guided IA PDRN injection	None (single-arm)	7 (could not walk on crutches painlessly)	0 (3-month follow-up; pain-free at 2 years 5 months)	Reduction to 0 with restored ROM (n = 1)	N/A — single-patient case report
*Ankle and foot*									
Kim and Chung (2015)/South Korea	RCT	Chronic plantar fasciitis	VAS	PDRN	Placebo (normal saline)	6.2 ± 1.5	3.7 ± 2.3 (12 weeks)	Significant (6.2→4.7 at 4 weeks p = 0.03; →3.7 at 12 weeks p = 0.002)	Significant at 12 weeks (PDRN 3.7 vs. placebo 6.3, p < 0.001); NS at 4 weeks
Lee et al. (2020)/South Korea	RCT	Plantar fasciitis	VAS	PDRN	Corticosteroid (triamcinolone 20 mg)	7.2 ± 1.7	3.5 ± 2.4 (6 months)	Significant at every visit (7.2→3.5)	PDRN worse than corticosteroid at 2 weeks (p = 0.010) and 6 weeks (p = 0.016); NS at 6 months (p = 0.523)
Lim et al. (2016)/South Korea	Case report	Posterior tibial tendon dysfunction	NRS	US-guided PDRN prolotherapy	None (single-arm)	8 (continuous medial ankle pain)	1 (sustained >1 year)	Gradual reduction (−7; no test, n = 1)	N/A — single-patient case report
*Spine*									
Kang et al. (2017)/South Korea	Case report	Lumbosacral radiculopathy with type 2 DM	NRS	Fluoroscopy-guided transforaminal epidural PDRN injection	None (single-arm)	8 (bedridden, severe radicular pain)	1 (sustained >6 months)	Gradual reduction (−7; no test, n = 1)	N/A — single-patient case report
Park (2017)/South Korea	Case report	Traumatic C5 nerve root injury	NRS	US-guided C5 nerve root block with PDRN	None (single-arm)	5 (neck/shoulder pain despite pregabalin)	∼0 (2 weeks after 2nd injection)	Gradual reduction (5→0; no test, n = 1)	N/A — single-patient case report
B. PN monotherapy									
*Knee joint*									
Moon et al. (2023)/South Korea	RCT	Knee OA	VAS (weight-bearing)	PN	HA; cross-linked HA	65.0 ± 16.4 (V1)	∼24.0 (V16)	Significant reduction (−41.0; p < 0.001)	Significant vs. HA (Δ −17.6; adj p = 0.048) and cross-linked HA (Δ −22.4; adj p = 0.016)
Vanelli et al. (2010)/Italy	RCT	Knee OA	VAS (global)	PN	HA injection	5.7 ± 1.9 (T0)	1.9 ± 1.5 (16 weeks)	Significant reduction (−3.8; p < 0.05)	N/A — no between-group test performed (control HA 4.9→2.1)
Kim T. W. et al. (2023)/South Korea	RCT	Knee OA	VAS (weight-bearing)	IA PN	IA HMWHA (Hyruan Plus)	N/R (change only)	N/R (change only)	PN −27.4 mm at 16 weeks (−54.0%; p < 0.001)	NS — vs. HMWHA change rate p = 0.296
Giarratana et al. (2014)/Italy	RCT	Knee OA	VAS	PN	HA (Hyalubrix)	N/R (graph only)	N/R (graph only)	PN significant from T2 (p = 0.043); T26 diff −1.33	Significant in favor of PN at T6 only
Kim T.W. et al. (2023)/South Korea	Observational study	Knee OA	VAS (100 mm)	PN	None (single-arm)	54.77 ± 19.59	25.00 ± 20.76 (6 months)	Significant (−29.77 mm, −54.4%; p < 0.0001)	N/A — single-arm observational
Kim et al. (2021a)/South Korea	Retrospective chart review	Knee OA	VAS (100 mm)	PN	HA; cross-linked HA	64.0 ± 18.2 (T0)	12.0 ± 13.0 (6 weeks)	−52.0 ± 16.4; significant within-group	Significant vs. HA (*post hoc* p = 0.006) and cross-linked HA (p = 0.029)
Mun et al. (2017)/South Korea	Case report	Knee OA	VAS	US-guided IAI PN injection	None (single-arm)	6 (refractory despite 6× prior HA)	1 (after 3rd injection, >5 months)	Gradual reduction (−5; no test, n = 1)	N/A — single-patient case report
C. PDRN/PN combination therapy									
*Temporomandibular joint*									
Cenzato et al. (2024)/Italy	RCT	TMJ osteoarthrosis	VAS	PN + HA, pericapsular	Physiotherapy (self-delivered exercises)	4.8 ± 2.87 (test, T0)	2.80 ± 2.78 (test, 12 weeks)	Significant (−41.7% at T3 vs. baseline; p = 0.006)	Significant — greater VAS decrease than physiotherapy at 12 weeks (p < 0.0001); baseline VAS imbalance (p < 0.001)
*Elbow joint*									
Shim et al. (2021)/South Korea	RCT	Lateral epicondylitis	VAS	EX + PDRN injection	EX; EX + ESWT	6.3 ± 1.5 (EX + PDRN)	2.3 ± 2.0 (12 weeks)	Significant reduction (−4.0; p < 0.001)	vs. EX only: significant at 12 weeks (p = 0.044); vs. EX + ESWT: NS (p = 0.452)
*Knee joint*									
Yoon et al. (2019)/South Korea	RCT	Knee OA	VAS	PDRN + HA injection	HA injection	71.27 ± 5.24 (baseline)	38.13 ± 4.47 (6 months)	Significant reduction (Δ −33.14 at 6 months)	Significant — greater than HA alone (control Δ −27.86; p = 0.0001)
Dallari et al. (2020)/Italy	RCT	Knee OA	WOMAC (pain subscale)	PN + HA	HA alone	N/R (graph only)	N/R (graph only)	Significant reduction T0–T3, T0–T4 (p < 0.0005), T0–T5 (p < 0.005)	NS (T3 p = 0.46, T4 p = 0.14, T5 p = 0.46)

ACL, anterior cruciate ligament; BCTQ, boston carpal tunnel questionnaire; DM, diabetes mellitus; ESWT, extracorporeal shockwave therapy; EX, exercise; HA, hyaluronic acid; HMWHA, high-molecular-weight hyaluronic acid; IA, intra-articular; LCL, lateral collateral ligament; MCID, minimal clinically important difference; N/A, not applicable; N/R, not reported; NRS, numeric rating scale; NS, not significant; OA, osteoarthritis; PDRN, polydeoxyribonucleotide; PN, polynucleotide; PT, physical therapy; RCT, randomized controlled trial; ROM, range of motion; TFCC, triangular fibrocartilage complex; TMD, temporomandibular disorder; TMJ, temporomandibular joint; US, ultrasound; VAS, visual analogue scale; WOMAC, Western Ontario and McMaster Universities Osteoarthritis Index.

Regarding functional disability, a study comparing PDRN + HA injection with HA injection in patients with knee osteoarthritis reported a statistically significant difference in the WOMAC used to assess knee function at 6 months (Yoon et al., 2019). In contrast, four studies reported improvement in pain and function, albeit without s significant between-group differences (Dallari et al., 2020; Kim J. Y. et al., 2021; Kim T. W. et al., 2023; Moon et al., 2023).

Three studies showed improvement in quality of life (EQ-5D), albeit without significant between-group differences (Kim J. Y. et al., 2021; Kim T. W. et al., 2023; Moon et al., 2023). Other related outcomes also showed an overall pattern of clinical improvement, but the between-group significance in the treatment versus control arms varied across studies. In traumatic C5 nerve root injury caused by cervical articular process fracture, ultrasound-guided PDRN injection elicited improvement in the NRS score (Park, 2017), while in lumbosacral radiculopathy, fluoroscopy-guided PDRN injection produced improvement in the NRS score (Kang et al., 2017).

#### 3.4.2 Preclinical effects of PDRN/PN

Histological and immunohistochemical analyses revealed that both PDRN and PN administration in shoulder rotator cuff models suppressed inflammatory M1 macrophages and activated regenerative M2 macrophages (Hwang et al., 2021; Seo et al., 2024). Furthermore, by promoting VEGF and proliferating cell nuclear antigen, PDRN and PN induced a dense, parallel arrangement of type I collagen (COL-1) at the tendon–bone junction and in tendon tissue (Kang et al., 2018; Kwon and Moon, 2020; Hwang et al., 2021; Kim D. H. et al., 2021; Joo et al., 2023), and significantly suppressed fatty infiltration and reduction in cross-sectional area (CSA) that accompany chronic injury (Hwang et al., 2021; Seo et al., 2024). In lumbar and nerve injury models, following the intervention, activation of the A2AR across the injury models induced neuronal survival and significantly suppressed tumor necrosis factor (TNF)-α and interleukin (IL)-1β (Joo et al., 2023). In addition, induction of VEGF expression improved perineural blood flow and significantly reduced axonal degeneration, as evidenced by neurofilament recovery and chromatolysis (Lee et al., 2021; Joo et al., 2023). Around the lumbar nerve roots, fibrosis was significantly lower in the intervention than in the corticosteroid control group (Lee et al., 2021). PDRN and combination therapy suppressed early neutrophil recruitment and local TNF-α and IL-6 across the ankle and Achilles tendon injury models (Rho et al., 2020; Kim D. S. et al., 2025), significantly increased the cAMP-PKA-CREB downstream signaling cascade beyond the levels of the control and normal groups, lowered caspase-3 and caspase-9 activity, and significantly suppressed the Bcl-2-associated X protein:B-cell lymphoma 2 expression ratio of the mitochondrial pathway, thereby blocking apoptosis of tenocytes and reversing structural degeneration (Rho et al., 2020). Moreover, it promoted organized arrangement of fibroblasts and COL-1 formation through phase-specific upregulation of VEGF, TGF-β1, and fibroblast growth factor (FGF) (Kang et al., 2018; Kim D. S. et al., 2025).

Biomechanical analyses revealed that PDRN and PN administration reinforced the attachment strength of the damaged or weakened tendon–bone junction in shoulder disorder models, significantly improving load-to-failure (Hwang et al., 2021; Kim D. H. et al., 2021; Seo et al., 2024), and when combined with extracorporeal shockwave or microcurrent therapy, exerted significant effects on the promotion of capillary network formation and thickness of the regenerated tendon (Kwon and Moon, 2020; Kim D. H. et al., 2021). In ankle models, administration of PDRN and combination therapy with atelocollagen significantly improved the CSA, resistance to mechanical stress, and stored energy (Kang et al., 2018; Rho et al., 2020; Kim D. S. et al., 2025). Combination with atelocollagen increased the tissue energy absorption capacity and reduced pain sensitivity to thermal stimulation (Rho et al., 2020).

Serological analyses showed that PDRN and PN administration significantly upregulated VEGF and FGF expression in shoulder disorder models, with PDRN showing a significantly greater effect at week 2 and PN at week 4 (Seo et al., 2024).

*In vivo* motion analyses revealed that administration of PDRN and combination therapy in shoulder models showed the fastest recovery and greatest improvement in locomotor function regarding walking distance, fast walking time, and walking speed (Seo et al., 2024). In lumbar and nerve injury models, it improved the sciatic functional index, alleviated neurogenic claudication (Joo et al., 2023), and significantly suppressed mechanical and thermal hyperalgesia in a dose-dependent manner (Figure 4) (Lee et al., 2021; Joo et al., 2023).

**FIGURE 4:**
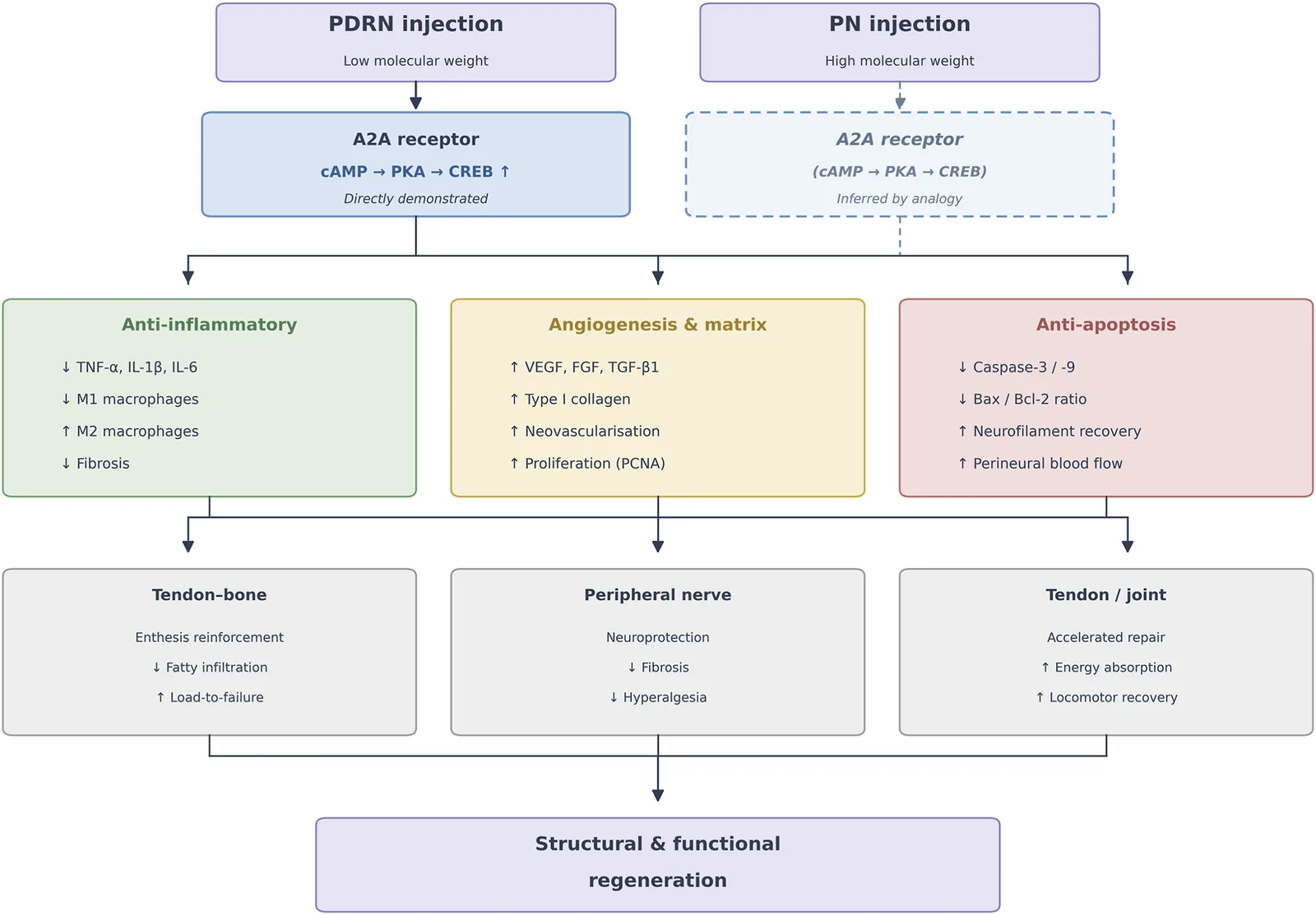
Preclinical mechanisms of PDRN/PN across musculoskeletal and nerve injury models. PDRN: polydeoxyribonucleotide, PN: polynucleotide.

### 3.5 Adverse events

Analysis of the safety of PDRN/PN showed that most clinical studies did not report serious adverse events directly attributable to the intervention. Specifically, some studies did not report adverse events at all—temporomandibular (n = 2) (Jee-Won Jang et al., 2025; Choi et al., 2026), elbow (n = 2) (Lee and Park, 2018; Shim et al., 2021), shoulder (n = 2) (Ryu et al., 2018; Kim et al., 2019), and wrist joints (n = 1) (Hyeon Joon Hong, 2025a) — whereas others explicitly reported that no adverse events occurred; (TMJ, n = 1 (Cenzato et al., 2024); shoulder, n = 2 (Yoon et al., 2017; Do et al., 2018); wrist, n = 2 (Park and Park, 2018; Hyeon Joon Hong, 2025b); spine, n = 1 (Park, 2017)). For the knee (n = 11), adverse events were reported but were mostly unrelated to the intervention. In nine studies, no adverse events occurred (Vanelli et al., 2010; Giarratana et al., 2014; Mun et al., 2017a; Mun et al., 2017b; Yoon et al., 2019; Dallari et al., 2020; Kim et al., 2020; Kim J. Y. et al., 2021; Moon et al., 2023). Among the reported cases, one study reported one case of mild and two cases of moderate adverse events, but no serious adverse events were reported (Kim W. H. et al., 2023); in another study, adverse events were reported but were unrelated to the intervention (Kim T. W. et al., 2023). In studies investigating the ankle/foot (n = 3), no intervention-related adverse effects were recorded (Kim and Chung, 2015; Lee et al., 2020), although one adverse effect associated with non-steroidal anti-inflammatory drugs was reported (Lim et al., 2016). The study on lumbar spinal joints reported no adverse events (Kang et al., 2017). Most preclinical studies did not separately report adverse events.

### 3.6 GRADE assessment of the included studies

The certainty of evidence for pain, assessed using GRADE, was low to very low across all comparisons supported by controlled evidence, with no comparison reaching moderate or high certainty (Table 4). Ratings were downgraded mainly for risk of bias, imprecision (most comparisons rested on a single small trial), and inconsistency across knee osteoarthritis trials.

**TABLE 4:** GRADE Summary of Findings for pain outcomes across comparisons supported by comparative evidence.

Comparison (site)	Outcome	Studies (design)	Effect summary	Risk of bias (RoB 2)	Certainty (GRADE)	Reasons for downgrading
Knee osteoarthritis						
PN vs. HA	Pain (VAS)	4 RCTs +1 retrospective (Moon 2023; Vanelli 2010; Giarratana 2014; Kim 2023; Kim 2021)	PN reduced pain more than HA in 3 studies; 1 RCT found no between-group difference (p = 0.296); 1 favored PN at a single time point	1 low (Moon 2023), 1 high (Kim 2023), others some concerns	⊕⊕○○ Low	Risk of bias (−1); inconsistency (−1: conflicting direction/significance across trials)
PDRN + HA or PN + HA vs. HA	Pain (VAS/WOMAC)	2 RCTs (Yoon 2019; Dallari 2020)	PDRN + HA superior to HA (p = 0.0001); PN + HA not significant vs. HA between groups	Some concerns (both)	⊕⊕○○ Low	Risk of bias (−1); inconsistency (−1: significant vs. non-significant between-group results)
Plantar fasciitis						
PDRN vs. placebo (saline)	Pain (VAS)	1 RCT (Kim 2015)	PDRN superior to placebo at 12 weeks (p < 0.001); NS at 4 weeks	Some concerns	⊕⊕○○ Low	Risk of bias (−1); imprecision (−1: single small RCT, n = 40)
PDRN vs. corticosteroid	Pain (VAS)	1 RCT (Lee 2020)	PDRN inferior to corticosteroid at 2 and 6 weeks; no difference at 6 months	Some concerns	⊕⊕○○ Low	Risk of bias (−1); imprecision (−1: single RCT; direction varies by time point)
Temporomandibular joint						
PN + HA vs. physiotherapy	Pain (VAS)	1 RCT (Cenzato 2024)	Greater VAS reduction than physiotherapy (p < 0.0001), but baseline VAS imbalance (p < 0.001)	High	⊕○○○ Very low	Risk of bias (−1: high, baseline imbalance); imprecision (−1: single RCT)
PDRN vs. hypertonic dextrose	Pain (VAS)	1 retrospective chart review, appraised as single-arm case series (Choi 2026)	Within-study before–after improvement in both agent subgroups; no concurrent control	JBI Case Series checklist: 9/10 (item 4 consecutive inclusion Unclear)	⊕○○○ Very low	Case-series design, no concurrent control (starts low); imprecision (−1: single small study)
Lateral epicondylitis						
Exercise + PDRN vs. exercise	Pain (VAS)	1 RCT (Shim 2021)	EX + PDRN superior to EX at 12 weeks (p = 0.044); NS vs. EX + ESWT	Some concerns	⊕⊕○○ Low	Risk of bias (−1); imprecision (−1: single RCT)

GRADE, certainty: ⊕⊕⊕⊕ high, ⊕⊕⊕○ moderate, ⊕⊕○○ low, ⊕○○○ very low.

ESWT, extracorporeal shockwave therapy; EX, exercise; GRADE, grading of recommendations assessment, Development and Evaluation; HA, hyaluronic acid; NS, not significant; OA, osteoarthritis; PDRN, polydeoxyribonucleotide; PN, polynucleotide; RCT, randomized controlled trial; RoB 2, Cochrane Risk of Bias 2 tool; VAS, visual analogue scale; WOMAC, Western Ontario and McMaster Universities Osteoarthritis Index.

## 4 Discussion

This systematic review reports that PDRN and PN interventions may have clinical and preclinical potential across a variety of joint and soft tissue disorders, based on 38 clinical and preclinical studies published over the past 17 years. Although the findings suggest potential improvements in pain and function with PDRN/PN treatment, the certainty of the clinical evidence remains limited, given the methodological weaknesses, heterogeneous study designs, and inconsistent between-group effects across the included studies.

In the clinical studies covered by this review, amelioration of pain (VAS/NRS) and improvements in function (WOMAC, MMO) were observed across the upper extremity joints, including the shoulder, elbow, and wrist, the TMJ, and lower extremity joints, including the knee, ankle, and foot; in particular, a consistent pain improvement effect was observed for the wrist across studies. However, between-group statistical significance for the intervention versus control varied by site and study. Even at sites of intensive research, such as the knee, a significant between-group difference was confirmed in only some studies, and between-group significance was not achieved for the quality-of-life (EQ-5D) outcome, despite an overall trend toward improvement.

In this review, PDRN acts via A2AR (Squadrito et al., 2017). Activation of the downstream A2AR–cAMP–PKA–CREB pathway by PDRN has been demonstrated at the protein level (cAMP, p-PKA, p-CREB) in an Achilles tendon-injury model (Rho et al., 2020), and partially corroborated at the cAMP–CREB level in a sciatic nerve-injury model (Joo et al., 2023). A2AR mediation of PDRN’s effect has additionally been confirmed pharmacologically, using the A2AR antagonist DMPX, in an epidural model of lumbar foraminal stenosis (Lee et al., 2021). For PN, this pathway was not directly examined; the included PN studies reported downstream indicators (e.g., growth factors, macrophage phenotype) rather than A2AR–cAMP-PKA-CREB signaling. Thus, the A2AR-mediated mechanism is directly established for PDRN and inferred by analogy for PN (Valls et al., 2009; Galeano et al., 2021).

Our findings demonstrated that PDRN/PN intervention improved biomechanical properties, such as the attachment strength of the tendon–bone junction and load-to-failure, and improved nerve regeneration and locomotor function. Load-to-failure is of considerable clinical significance since it is a direct indicator of attenuated re-rupture risk in the treatment of tendon injury.

Similarly, previous studies have reported that PDRN plus atelocollagen effectively promoted healing of the bone–tendon junction, showed better recovery in biomechanical and morphological aspects, and increased COL-1 expression in a rabbit model of chronic rotator cuff tear, thereby inducing early functional recovery (Jo et al., 2023). The finding of this review, viz. PDRN plus atelocollagen improved the CSA, resistance to mechanical stress, and stored energy, is consistent with that of prior studies (Kim D. S. et al., 2025).

PDRN and PN share an A2AR-mediated regenerative mechanism, but show distinct characteristics in the temporal profile of growth factor expression and cellular and tissue responses owing to differences in molecular weight and physical structure. Low-molecular-weight PDRN significantly upregulates VEGF and FGF expression in the early phase (week 2), whereas high-molecular-weight PN elicits similar effects in the later phase (week 4). Seo et al. also reported that plasma VEGF and FGF increased in both the PDRN and PN administration groups in a diabetic chronic rotator cuff tear rat model, and that high-molecular-weight PN tended to maintain relatively prolonged growth factor expression (Seo et al., 2024). This can be attributed to differences in physical properties: low-molecular-weight PDRN diffuses rapidly within the tissue and induces early regenerative signaling, whereas high-molecular-weight PN forms a three-dimensional scaffold structure and is released slowly; therefore, its effect persists and is expressed for a protracted period.

Huang et al. detailed the response patterns of the two agents at the cellular and tissue levels in a rotator cuff tear rat model. At 6 weeks post-intervention, reduction in CD68-positive cells, a marker of inflammatory macrophages was observed in both the PDRN repair (PR) and PN repair (PNR) groups compared with the saline (SR) group, confirming a shift toward inflammation resolution and regeneration; however, because the PR group retained slightly more CD68-positive cells than the PNR group, PN showed a relative advantage in the resolution of inflammatory macrophages. In contrast, CD168-positive cells, a marker of the regenerative macrophage lineage, were more abundant in the PR and PNR groups than in the SR group at 3 weeks after repair and were most abundant in the PR group; the muscle fiber CSA was also greater in the PR group versus the SR and PNR groups, implying that PDRN played a prominent role in eliciting an early regenerative response and recovery from muscle atrophy (Hwang et al., 2021). Preclinical evidence, although limited, suggests that PDRN and PN may differ in the temporal dynamics of their regenerative actions (Hwang et al., 2021; Seo et al., 2024). The rapid tissue diffusion of PDRN may favor an early regenerative response, whereas the scaffold-forming properties of PN may support a more gradual and prolonged response. These distinct profiles raise the possibility that the two agents serve complementary therapeutic roles, with PDRN better suited to conditions requiring early regeneration and PN to those requiring sustained structural support. This interpretation, however, is hypothesis-generating rather than clinically validated, as it derives from a small number of preclinical models and has not been tested in direct comparative studies. It should therefore be regarded as a direction for future research rather than an established criterion for agent selection, and its clinical relevance will need to be clarified through adequately powered head-to-head trials.

No major safety signal attributable to PDRN/PN was identified in the available clinical studies, however, given the incomplete and heterogeneous adverse event reporting, their safety profile warrants further confirmation.

Most clinical studies included in this review employed an ultrasound-guided injection technique: fluoroscopy-guided epidural injection was performed for lumbar neuropathy and ultrasound-guided nerve root block for cervical nerve root injury for spinal disorders. The standard use of image-guided techniques shows that, in recent musculoskeletal interventions, ultrasound guidance enhances accuracy and safety. In a scoping review of ultrasound-guided acupuncture, Lee et al. reported that ultrasound guidance improves needle placement accuracy and procedural safety through real-time anatomical confirmation, generally showing significant improvements in pain and functional outcomes (Lee et al., 2024). Moreover, in a systematic review and meta-analysis of ultrasound-guided pharmacopuncture, Lee et al. reported that the ultrasound-guided group had a significantly higher proportion of patients with pain reduction and fewer reported adverse events than the non-guided group (particularly marked in lumbar disorders) (Lee et al., 2025). This prior evidence aligns with the finding in this review that PDRN/PN injections performed under ultrasound or fluoroscopy guidance ameliorated pain and improved function without serious adverse events.

Tendon disorders of specific joints were frequently reported in this review, and the conservative treatments commonly documented included physiotherapy (Khan and Scott, 2009), extracorporeal shockwave therapy (Wang et al., 2003), corticosteroid injection (Coombes et al., 2010), and hyaluronic acid (Altman et al., 2015); prior studies have further reported collagen injection (Chirico et al., 2026) and platelet-rich plasma (Chen et al., 2018; Kale et al., 2024) as widely used options (Table 5) (Flower and Blackwell, 1979; Langberg et al., 2007; Notarnicola and Moretti, 2012; Kim J. H. et al., 2025).

**TABLE 5:** Commonly used treatments for tendinopathy and related musculoskeletal disorders.

Treatment	Proposed mechanism	Common indications	Included studies
Physiotherapy (exercise)	Progressive mechanical loading promoting tendon remodeling and load tolerance (Khan and Scott, 2009)	First-line for most chronic tendinopathies (e.g., rotator cuff, lateral epicondylitis)	TMJ Osteoarthrosis (Cenzato et al., 2024), Lateral epicondylitis (Shim et al., 2021)
Corticosteroid injection	Local anti-inflammatory action reducing pain and swelling (Coombes et al., 2010)	Short-term relief of tendon/joint pain	Plantar fasciitis (Lee et al., 2020)
Extracorporeal shockwave therapy (ESWT)	Mechanotransduction stimulating neovascularization and tissue repair (Wang et al., 2003)	Calcific/chronic tendinopathy unresponsive to conservative care	Lateral epicondylitis (Shim et al., 2021)
Collagen injection	Supplies structural matrix to support tendon/ligament repair (Chirico et al., 2026)	Adjunct in tendinopathy and soft-tissue injury	N/R
Platelet-rich plasma (PRP)	Delivers autologous growth factors to promote healing (Kale et al., 2024)	Chronic tendinopathy, osteoarthritis	N/R
Hyaluronic acid (HA) injection	Restores the viscoelasticity of synovial fluid and provides cartilage protection (cushion effect) and lubrication, relieving pain (viscosupplementation) (Altman et al., 2015)	Osteoarthritis (e.g., knee), tendinopathy	Knee osteoarthritis (Moon et al., 2023; Vanelli et al., 2010; Kim T. W. et al., 2023; Kim et al., 2021; Giarratana et al., 2014; Yoon et al., 2019; Dallari et al., 2020)
Polydeoxyribonucleotide (PDRN)/Polynucleotide (PN)	Adenosine A2A receptor–mediated anti-inflammatory and tissue-regenerative effects; viscosupplementation (PN) with growth-factor/angiogenic stimulation	Arthritis, tendinopathy, and soft tissue or nerve-related pain	See Table 1

This study is distinct from prior reviews because it provides integrated evidence encompassing both PDRN and PN interventions. While prior studies were confined to PDRN monotherapy (Kim et al., 2024; Nam et al., 2025), a specific condition (e.g., tendon disorders) (Luo et al., 2025), or summarizing the clinical status, this study encompassed a variety of joint sites and musculoskeletal neurological disorders beyond tendon disorders, searched 10 databases in accordance with the PRISMA guidelines, and formally assessed the quality of clinical and preclinical studies using the Cochrane RoB 2 and SYRCLE tools, respectively. It systematically presented mechanism-effect evidence by rigorously distinguishing between PDRN and PN interventions used interchangeably in clinical practice, and demonstrating that molecular-level mechanisms elucidated in preclinical studies—such as A2AR activation, M2 macrophage (CD163/168) differentiation, and blockade of apoptosis (caspase-3/9)—translate into clinical functional recovery (WOMAC, MMO) and increased biomechanical load-to-failure.

This study has some limitations. First, the substantial heterogeneity among the included studies in terms of disease site, mode of intervention (monotherapy/combination, use of ultrasound guidance), control group (HA, corticosteroid, placebo, exercise therapy, etc.), and outcome measures precluded a quantitative meta-analysis, and only a qualitative synthesis could be conducted. Second, the marked geographic concentration limited generalizability of the results. Despite searching 10 databases without language restrictions, 24 (86%) of 28 clinical studies were published in Korea, and 4 (14%) in Italy, and all 10 preclinical studies were conducted in Korea; thus, these two countries predominated research in this field. This appears to stem from the fact that, following the initial approval of tissue regenerative agents in Italy (Thellung et al., 1999), Korea expanded its application to musculoskeletal research based on domestic production and establishment of a mass production system for regenerative agents (Kim et al., 2024). Third, the quality of the evidence was limited. In RoB 2 assessment of 10 clinical RCTs, only one study was classified as “low risk,” two as “high risk,” and the remainder as having “some concerns.” The main reasons were omission of the description of the allocation concealment method, the possibility of bias in subjective outcomes due to the single-blind design that is unavoidable given the nature of injection interventions, and concern about selective reporting owing to unverified prospective protocol registration. Similarly, SYRCLE assessment of the 10 preclinical RCTs showed high transparency of outcome reporting, although many received an “unclear” rating owing to the lack of descriptions of items related to random sequence generation, allocation, concealment, and blinding. Fourth, the level of clinical evidence was limited relative to the preclinical mechanistic evidence. In particular, many knee studies used the effective active comparator HA as the control (five knee RCTs were superiority trials (Vanelli et al., 2010; Yoon et al., 2019; Dallari et al., 2020; Kim T. W. et al., 2023; Moon et al., 2023) and 1 an equivalence trial (Giarratana et al., 2014)), and the lack of a significant between-group difference observed in some studies may stem from the efficacy of the comparator and study design rather than the low efficacy of the intervention. This interpretive difficulty is compounded by a regulatory distinction between the two agents. Classification hinges on a product’s principal mode of action; under the European framework, a pharmacological, immunological, or metabolic action qualifies a product as a medicinal product (Directive 2001/83/EC), whereas a primarily physical or mechanical action qualifies it as a substance-based medical device (Regulation (EU) 2017/745). PDRN, acting through receptor-mediated biological activity, is generally regulated as a medicinal product, whereas several intra-articular PN preparations are marketed as Conformité Européenne (CE)-marked class III medical devices on the basis of their physical, viscoelastic mode of action—a device pathway also used in Korea for viscosupplementation in knee osteoarthritis. Because device and medicinal frameworks impose different pre-market clinical evidence requirements, and because PN’s device status places it in the same regulatory category as HA viscosupplements, several included knee trials were designed as superiority or equivalence trials against HA rather than placebo. This regulatory context therefore partly explains both the heterogeneity of comparators across the included studies and the design-driven, rather than efficacy-driven, absence of between-group differences noted above (AIFA, 2026; MFDS, 2026).

However, since these design characteristics do not directly demonstrate efficacy, caution is required in interpretation. Fifth, the safety evidence was marred by asymmetry.

Although no serious adverse events directly related to the intervention were reported in clinical studies, because most preclinical studies did not designate adverse events as a separate outcome measure or did not evaluate and report them, information on the safety of the intervention could not be ascertained.

As the certainty of evidence for pain was low to very low across all controlled comparisons, the apparent benefits of PDRN and PN should be regarded as preliminary; conclusions from randomized, retrospective, and single-arm studies cannot be weighted equally, and adequately powered, low-risk-of-bias trials are needed before these agents can be recommended for specific indications.

We propose the following directions for future research: First, large-scale RCTs with enhanced methodological rigor are needed, with clearly described allocation concealment, rigorous blinding of the outcome assessor even when patient blinding is unfeasible, and prospective protocol registration. Second, studies directly comparing PDRN and PN are warranted to define the optimal molecular weight-based indications by site and disease (e.g., PDRN for tendon/ligament lesions and PN for weight-bearing joints), with standardized dosing and injection routes reported according to the CONSORT guidelines. Third, translational designs that longitudinally track ultrasound-/MRI-based structural outcomes and quality of life (EQ-5D) beyond pain and functional outcomes are required. Fourth, factorial design studies should verify the synergistic effects of combination therapies, including extracorporeal shockwave therapy, microcurrent, and atelocollagen. Multinational studies in regions beyond Korea and Italy remain warranted to enhance the generalizability of the results.

## 5 Conclusion

In conclusion, available evidence suggests potential improvements in pain and function following PDRN/PN treatment; however, the certainty of the evidence remains limited because of methodological limitations, heterogeneous study designs, and inconsistent comparative effects. In preclinical studies, both agents induced anti-inflammatory, angiogenic, and anti-apoptotic effects, as well as COL-1 formation through the A2AR signaling pathway, which translated into reinforcement of the tendon–bone junction and recovery of locomotor function. No serious adverse events directly related to the intervention were reported across clinical studies and no major safety signal attributable to PDRN/PN was identified; however, the safety evidence remains limited by incomplete and heterogeneous adverse event reporting, and confirmation through trials with standardized safety monitoring is warranted. Nonetheless, given the limitations in the quality of the clinical evidence and geographic representativeness, verification through methodologically rigorous, prospectively registered, large-scale multinational randomized controlled trials is warranted.

## Data Availability

The original contributions presented in the study are included in the article/Supplementary Material, further inquiries can be directed to the corresponding author.
