# Supplementary material for: Polydeoxyribonucleotide and polynucleotide effects in musculoskeletal disorders: a systematic review
Source: Front Pharmacol. 2026 Oct 28;17:1965494. doi: 10.3389/fphar.2026.1965494 (PMC13752129; doi:10.3389/fphar.2026.1965494)
Supplement: Supplementary file 1 [file Table1.docx]

**Supplementary Material**

**Supplementary Table 1. Search strategy**

| **Ovid-Medline 1946 to March 2026** | | **Date: 2026.03.26** |
| --- | --- | --- |
| Searches | | Results |
| 1 | Polydeoxyribonucleotides.mp. | 2828 |
| 2 | Polynucleotides.mp. | 7218 |
| 3 | PDRN.mp. | 247 |
| 4 | Placentex.mp. | 7 |
| 5 | or/1-4 | 9790 |

| **EMBASE 1974 to 2026 March 24** | | **Date: 2026.03.26** |
| --- | --- | --- |
| Searches | | Results |
| 1 | Polydeoxyribonucleotides.mp. | 217 |
| 2 | Polynucleotides.mp. | 2546 |
| 3 | PDRN.mp. | 313 |
| 4 | Placentex.mp. | 31 |
| 5 | or/1-4 | 2979 |

| **Ovid-AMED 1985 to February 2026** | | **Date: 2026.03.26** |
| --- | --- | --- |
| Searches | | Results |
| 1 | Polydeoxyribonucleotides.mp. | 1 |
| 2 | Polynucleotides.mp. | 0 |
| 3 | PDRN.mp. | 1 |
| 4 | Placentex.mp. | 0 |
| 5 | or/1-4 | 1 |

| **Cochrane Library** | | **Date: 2026.03.26** |
| --- | --- | --- |
| Searches | | Results |
| 1 | Polydeoxyribonucleotides | 104 |
| 2 | Polynucleotides | 3 |
| 3 | PDRN | 175 |
| 4 | #1 or #2 or #3 | 175 |

**China database**

| **CHINA Academic Journals Full-text Database** | **Date: 2026.03.26** |
| --- | --- |
| Searches | Results |
| (SU=‘Polydeoxyribonucleotides’ OR SU=‘Polynucleotides’ OR SU=’PDRN’) | 26 |
| (SU=‘多聚脱氧核糖核苷酸’ OR SU=‘多核苷酸’) | 74 |

**Japan database**

| CiNii Research (https://ci.nii.ac.jp/) | Date: 2026.03.26 |
| --- | --- |
| Searches | Results |
| (Polydeoxyribonucleotides OR Polynucleotides OR PDRN) AND (injection) | 30 |

**Korean databases**

| **KoreaMed (https://koreamed.org/SearchBasic.php)** | **Date: 2026.03.26** |
| --- | --- |
| Searches | Results |
| Polydeoxyribonucleotides OR Polynucleotides OR PDRN | 52 |

| **KMbase (https://kmbase.medric.or.kr/)** | **Date: 2026.03.26** |
| --- | --- |
| Searches | Results |
| Polydeoxyribonucleotides | 2 |
| Polynucleotides | 1 |
| PDRN | 1 |

| **OASIS (https://oasis.kiom.re.kr/)** | **Date: 2026.03.26** |
| --- | --- |
| Searches | Results |
| Polydeoxyribonucleotides | 4 |
| Polynucleotides | 0 |
| PDRN | 3 |

| ScienceON (https://scienceon.kisti.re.kr/srch/selectPORSrchArticleList.do) | Date: 2026.03.26 |
| --- | --- |
| Searches | Results |
| (Polydeoxyribonucleotides OR Polynucleotides OR PDRN) AND (injection) | 127 |

**Supplementary Table 2.**

**Methodological quality (risk of bias) assessment of the included studies, using design-specific appraisal tools.**

**A. Non-randomised comparative studies — ROBINS-I**

| **Study** | **D1** | **D2** | **D3** | **D4** | **D5** | **D6** | **D7** | **Overall** |
| --- | --- | --- | --- | --- | --- | --- | --- | --- |
| Yoon 2017 | Serious | Moderate | Low | Moderate | Moderate | Moderate | Moderate | Serious |
| Kim 2021 | Serious | Moderate | Low | Serious | Moderate | Moderate | Moderate | Serious |
| ***D1****, bias due to confounding;* ***D2****, bias in selection of participants into the study;* ***D3****, bias in classification of interventions;* ***D4****, bias due to deviations from intended interventions;* ***D5****, bias due to missing data;* ***D6****, bias in measurement of outcomes;* ***D7****, bias in selection of the reported result.* ***Judgements****: Low / Moderate / Serious / Critical / No information.* | | | | | | | | |

**B. Single-arm before–after studies — NIH Quality Assessment Tool for Before-After (Pre-Post) Studies With No Control Group**

| **Study** | **1** | **2** | **3** | **4** | **5** | **6** | **7** | **8** | **9** | **10** | **11** | **12** | **Overall** |
| --- | --- | --- | --- | --- | --- | --- | --- | --- | --- | --- | --- | --- | --- |
| Do 2018 | Yes | Yes | Yes | Yes | No | Yes | Yes | No | Yes | Yes | No | NA | Fair |
| Kim 2023 | Yes | Yes | Yes | CD | Yes | Yes | Yes | No | Yes | Yes | No | NA | Fair |
| *Items: 1, study question/objective clearly stated; 2, eligibility/selection criteria prespecified and clearly described; 3, participants representative of the eligible population; 4, all eligible participants enrolled; 5, sample size sufficiently large; 6, intervention clearly described and delivered consistently; 7, outcome measures prespecified, valid, reliable, and assessed consistently; 8, outcome assessors blinded to interventions; 9, loss to follow-up 20% or less and accounted for; 10, statistical tests providing p values for pre-to-post changes; 11, outcomes measured multiple times before and after the intervention; 12, group-level intervention analysed with individual-level data. Rating: Yes / No / CD (cannot determine) / NR (not reported) / NA (not applicable). Overall rating (Good / Fair / Poor) reflects risk of bias per NIH guidance, not a numeric cutoff of Yes answers.* | | | | | | | | | | | | | |

**C. Single-arm studies (case series) — JBI Critical Appraisal Checklist for Case Series (10 items)**

| **Study** | **1** | **2** | **3** | **4** | **5** | **6** | **7** | **8** | **9** | **10** | **Summary** |
| --- | --- | --- | --- | --- | --- | --- | --- | --- | --- | --- | --- |
| Ryu 2018 | Yes | Yes | Yes | Unclear | Unclear | Yes | Yes | Yes | Yes | Yes | Yes 8 / No 0 / Unclear 2 / NA 0 |
| Lee 2018 | Unclear | Yes | Yes | Unclear | Unclear | Yes | Yes | Yes | Yes | NA | Yes 6 / No 0 / Unclear 3 / NA 1 |
| Jang 2025 | Yes | Yes | Yes | Unclear | Yes | Yes | Yes | Yes | Yes | Yes | Yes 9 / No 0 / Unclear 1 / NA 0 |
| Hong 2025 (wrist) | Yes | Yes | Unclear | Unclear | Unclear | Yes | Yes | Yes | Yes | NA | Yes 6 / No 0 / Unclear 3 / NA 1 |
| Choi 2026 | Yes | Yes | Yes | Unclear | Yes | Yes | Yes | Yes | Yes | Yes | Yes 9 / No 0 / Unclear 1 / NA 0 |
| *Items: 1, clear inclusion criteria; 2, condition measured in a standard, reliable way; 3, valid methods for identification of the condition; 4, consecutive inclusion of participants; 5, complete inclusion of participants; 6, clear reporting of demographics; 7, clear reporting of clinical information; 8, outcomes or follow-up results clearly reported; 9, clear reporting of presenting site(s)/clinic(s) demographic information; 10, statistical analysis appropriate. Rating: Yes / No / Unclear / NA (not applicable).* | | | | | | | | | | | |
| ***Note:*** *Choi 2026, Jang 2025, Do 2018 and Kim 2023 contain between-agent or single-arm before–after comparisons but were appraised as single-arm case series, as no independent (concurrent) control group was included.* | | | | | | | | | | | |

**D. Single-patient reports (case reports) — JBI Critical Appraisal Checklist for Case Reports (8 items)**

| **Study** | **1** | **2** | **3** | **4** | **5** | **6** | **7** | **8** | **Summary** |
| --- | --- | --- | --- | --- | --- | --- | --- | --- | --- |
| Kim 2019 | Yes | Yes | Yes | Yes | Yes | Yes | Yes | Yes | Yes 8 / No 0 / Unclear 0 / NA 0 |
| Park 2018 | Yes | Yes | Yes | Yes | Yes | Yes | Yes | Yes | Yes 8 / No 0 / Unclear 0 / NA 0 |
| Hong 2025 (TFCC) | Yes | Yes | Yes | Yes | Yes | Yes | Unclear | Yes | Yes 7 / No 0 / Unclear 1 / NA 0 |
| Mun 2017 (KOA) | Yes | Yes | Yes | Yes | Yes | Yes | Yes | Yes | Yes 8 / No 0 / Unclear 0 / NA 0 |
| Mun 2017 (PA) | Yes | Yes | Yes | Yes | Yes | Yes | Yes | Yes | Yes 8 / No 0 / Unclear 0 / NA 0 |
| Kim 2020 | Yes | Yes | Yes | Yes | Yes | Yes | Yes | Yes | Yes 8 / No 0 / Unclear 0 / NA 0 |
| Lim 2016 | Yes | Yes | Yes | Yes | Yes | Yes | Yes | Yes | Yes 8 / No 0 / Unclear 0 / NA 0 |
| Kang 2017 | Yes | Yes | Yes | Yes | Yes | Yes | Yes | Yes | Yes 8 / No 0 / Unclear 0 / NA 0 |
| Park 2017 | Yes | Yes | Yes | Yes | Yes | Yes | Yes | Yes | Yes 8 / No 0 / Unclear 0 / NA 0 |
| *Items: 1, patient's demographic characteristics clearly described; 2, patient's history clearly described and presented as a timeline; 3, current clinical condition on presentation clearly described; 4, diagnostic tests or assessment methods and results clearly described; 5, intervention(s) or treatment procedure(s) clearly described; 6, post-intervention clinical condition clearly described; 7, adverse events (harms) or unanticipated events identified and described; 8, takeaway lessons provided. Rating: Yes / No / Unclear / NA (not applicable).* | | | | | | | | | |
| ***Note: all nine studies are single-patient reports; the JBI Case Reports tool (8 items) therefore applies rather than the Case Series tool (10 items). KOA, knee osteoarthritis; PA, pes anserine bursitis; TFCC, triangular fibrocartilage complex.*** | | | | | | | | | |
